# Relationship between visual field loss and contrast threshold elevation in glaucoma

**DOI:** 10.1186/1471-2415-5-22

**Published:** 2005-09-13

**Authors:** CM Tochel, JS Morton, JL Jay, JD Morrison

**Affiliations:** 1 Institute of Biomedical & Life Sciences, West Medical Building, University of Glasgow, Glasgow, Scotland, UK; 2 Tennent Institute of Ophthalmology, Gartnavel General Hospital, 1053 Great Western, Road, Glasgow, Scotland, UK

## Abstract

**Background:**

There is a considerable body of literature which indicates that contrast thresholds for the detection of sinusoidal grating patterns are abnormally high in glaucoma, though just how these elevations are related to the location of visual field loss remains unknown. Our aim, therefore, has been to determine the relationship between contrast threshold elevation and visual field loss in corresponding regions of the peripheral visual field in glaucoma patients.

**Methods:**

Contrast thresholds were measured in arcuate regions of the superior, inferior, nasal and temporal visual field in response to laser interference fringes presented in the Maxwellian view. The display consisted of vertical green stationary laser interference fringes of spatial frequency 1.0 c deg^-1 ^which appeared in a rotatable viewing area in the form of a truncated quadrant extending from 10 to 20° from fixation which was marked with a central fixation light. Results were obtained from 36 normal control subjects in order to provide a normal reference for 21 glaucoma patients and 5 OHT (ocular hypertensive) patients for whom full clinical data, including Friedmann visual fields, had been obtained.

**Results:**

Abnormally high contrast thresholds were identified in 20 out of 21 glaucoma patients and in 2 out of 5 OHT patients when compared with the 95% upper prediction limit for normal values from one eye of the 36 normal age-matched control subjects. Additionally, inter-ocular differences in contrast threshold were also abnormally high in 18 out of 20 glaucoma patients who had vision in both eyes compared with the 95% upper prediction limit. Correspondence between abnormally high contrast thresholds and visual field loss in the truncated quadrants was significant in 5 patients, borderline in 4 patients and absent in 9 patients.

**Conclusion:**

While the glaucoma patients tested in our study invariably had abnormally high contrast thresholds in one or more of the truncated quadrants in at least one eye, reasonable correspondence with the location of the visual field loss only occurred in half the patients studied. Hence, while contrast threshold elevations are indicative of glaucomatous damage to vision, they are providing a different assessment of visual function from conventional visual field tests.

## Background

The visual field loss caused by glaucoma has been divided into 5 stages ranging from relative defects in sensitivity to almost complete visual loss [1]. The pattern of loss which alerts awareness of the disease, however, is often an arcuate scotoma occurring with approximately equal frequency in the superior or inferior hemifield within 20 degrees of the fovea and with or without a nasal step. These losses are generally detected in the clinical environment by visual field tests involving presentation of spots of light within a hemi-spherical dome at various locations within the central visual field. However, by the time visual field defects are detected, considerable loss of retinal ganglion cells may already have occurred, as exemplified by the case of a glaucoma suspect who, despite an absence of local field defects prior to death, showed ganglion cell losses *post-mortem *of 63% and 44% in left and right retinae, respectively [2]. Accordingly, there has been an impetus to develop alternative tests to visual field testing capable of detecting glaucoma at earlier stages. One such test is the measurement of contrast sensitivity in response to sinusoidal grating patterns generated by a variety of methods-oscilloscope, TV or computer monitor, printed paper, laser interferometry, or Snellen letters. The most usual protocol has involved measurements of contrast sensitivity in response to foveal viewing of the display [3-14], though more involved studies have additionally determined contrast sensitivities at peripheral locations in the visual field [15-22]. The consensus has been, with one exception [13], that contrast sensitivity in response to low spatial frequencies is impaired in glaucoma, leading to strong advocacy of its usefulness in glaucoma screening. While most studies reported the results of visual field tests, correlations between impaired contrast sensitivity and the location of visual field loss were generally not undertaken. In the one case in which this was done on a limited scale [17], contrast sensitivity deficits were described as occurring in both the visual hemifield showing no visual loss and the hemifield which did show visual loss.

We have therefore set out to answer the question whether regions of glaucomatous visual field loss are associated with elevations of contrast threshold which was employed rather than its reciprocal, the contrast sensitivity. For this purpose, we have designed a streamlined method of measuring contrast thresholds at different peripheral locations in the visual field based on the advantages offered by viewing laser interference fringes in the Maxwellian view. This provides a large visual field which can be viewed without the need for refraction of the viewer and has a sense of proximity, rather than the unavoidable remoteness of an externally generated grating display. Our results were then compared with visual field data obtained in the conventional manner. A preliminary report of our results has been made previously [23].

## Methods

### Patients

The experiments were undertaken with the informed consent of the participants and with the approval of the Ethics Committee of the North Glasgow University Hospitals NHS Trust and were performed in conformity with the Declaration of Helsinki. All subjects and patients underwent the Snellen test to determine their best visual acuity with refraction if necessary.

A total of 26 patients ages 37 to 83 yr (mean 69 ± 10 S.D. yr) were recruited from the Tennent Institute of Ophthalmology, Gartnavel General Hospital. They were diagnosed according to their intra-ocular pressure (IOP) determined by applanation tonometry, the appearance of the optic disc on ophthalmoscopic examination and the extent of visual field loss determined with the Friedmann Visual Field Analyzer Mark II (Clement Clarke International Ltd., London). Twenty one patients were diagnosed as having glaucoma (Table 1): the most common condition was primary open angle glaucoma (POAG) in which IOP exceeded 22 mmHg on at least 2 occasions before treatment, there was cupping of the optic disc and visual field defects characteristic of glaucoma were present. Normal pressure glaucoma (NPG) was diagnosed according to the same criteria as POAG except that IOP was less than 22 mmHg. In addition, one patient had each of primary chronic angle closure glaucoma, post-traumatic glaucoma and sarcoid-induced glaucoma. Within the glaucoma group, since 6 patients had a fellow eye which showed no visual field defects or definite pathological cupping of the optic disc and had an IOP of less than 22 mmHg, these eyes were designated normal, though were not used as controls. The 5 ocular hypertensives (OHT) were defined on the basis of an IOP which exceeded 21 mmHg but with no visual field loss and were subdivided into those with and without pathological cupping of the optic disc (Table 1). All patients were selected on the basis of having stable visual fields through being superimposable in terms of their position, extent and depth, tested usually on a six monthly basis over the 2 years preceding the study. Five patients who were subsequently retested over intervals of 1 day to 6 months showed reproducible visual fields.

As controls, we examined 36 non-glaucoma subjects ages 48–78 yr (mean 62 ± 8 S.D. yr.) who were recruited from within the University and from personal acquaintances. They were deemed to be normal on the basis of a reported absence of visual problems, a Snellen acuity of 6/6 or better in each eye and a report of normal vision from a recent visit to their optometrist. While 28 subjects had 2 normal eyes, 8 subjects had one normal eye and a non-glaucomatous problem in the fellow eye, which is dealt with separately. Of the 36 subjects, 19 were emmetropic while the remainder had refractive errors ranging from +1.50 DS to -7.00 DS with astigmatism of up to +5.00 DC and all were presbyopic to varying extents. Twenty three subjects ages 55–78 yr, including all subjects above 69 yr, were also examined with the Central 24-2 threshold test with the Humphrey II Visual Field Analyzer model 750 (Humphrey Instruments, San Leandro, CA, USA) to which we had ready access at times convenient to our subjects.

### Apparatus

**Figure 1:**
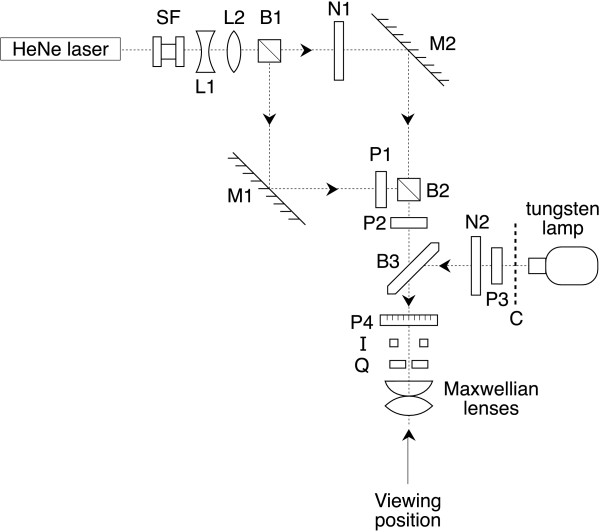
Schematic representation of optical apparatus used to generate vertical sinusoidal laser interference fringes which are observed in the Maxwellian view. Abbreviations: A aperture, B beam splitter, C interference filter, I iris diaphragm, L lens, M front silvered mirror, N neutral density filter, P polarizer, SF spatial filter. Further explanation is given in Methods.

**Figure 2:**
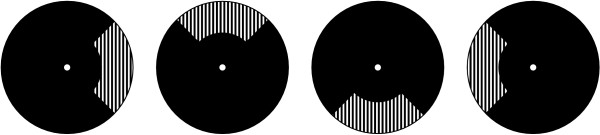
Schematic representation of the truncated quadrants in the temporal, superior, inferior and nasal positions as seen by the right eye. The central fixation light was of subtense 2 deg and the truncated quadrant extended from 10–20 deg. The sinusoidal interference fringe pattern had a spatial frequency of 1.0 c deg^-1^.

Contrast thresholds were measured with a modification of a laser interferometer described previously [24,25] and is shown schematically in Figure 1. Monochromatic green light (λ = 543 nm) from a 0.95 mW He-Ne laser (Uniphase 1652P) was passed through a spatial filter (SF), a -10 DS lens (L1) to expand the beam and a +10 DS lens (L2) to collimate the beam. The beam was then divided by a 2 inch cube beamsplitter (B1), the 2 beams reflected from λ/20 front silvered mirrors (M1 and M2) and recombined by a second 2 inch cube beamsplitter (B2). The two beams were equated in intensity by the neutral density filter N1 and the polarization shift caused by the beamsplitters was compensated by the rotatable sheet polarizer (P1, extinction = 10^-4^). The combined beams were passed through a second polarizer (P2) to sharpen the polarization prior to combination at the plate beamsplitter B3 with the background beam which consisted of non-coherent light from a tungsten filament microscope lamp. The latter beam was passed through heat absorbing filters, a green interference filter (C, peak λ = 546 nm), a polarizer (P3) to polarize the light at 90° to the laser beams and a neutral density filter (N2) to equate the intensity to that of the laser beams. The combined laser and background beams were passed through a rotatable polarizer of 2 inch diameter (P4, Coherent-Ealing 22-9161). The emergent beam passed through an iris diaphragm (I) which controlled the overall field size, an aperture consisting of a rotatable truncated quadrant (Q) and the Maxwellian lens assembly which consisted of 38 mm diameter +20 DS plano-convex and +32 DS biconvex high refractive index lenses mounted in the same housing as the rotatable aperture. The total power of the Maxwellian lens assembly was +42 DS. The subject or patient looked into the Maxwellian lens with the chin supported by a chin rest. The observed visual display consisted of an attenuated green central fixation light of subtense 2° and the truncated quadrant which was rotatable to the 4 chosen visual field positions *viz*. temporal, superior, inferior and nasal, as shown for right eye viewing in Figure 2.

The truncated quadrant extended from 10° to 20° from the central fixation point. The angular dimensions were calibrated from determination of the location of the nasal and temporal margins of the blind spot from the axis of fixation. These were determined first in angular subtenses for viewing a sheet of graph paper from 25 cm. The linear dimensions of the blind spot from the axis of fixation were then obtained for viewing a needle mounted on a micrometer, coplanar with the iris diaphragm, through the Maxwellian lens. The factor to convert linear dimensions into angular dimensions was then used to convert the linear dimension of the Maxwellian display into angular subtenses. The procedure was undertaken in both eyes in 3 subjects with close agreement between the results.

By translation of mirror M1 to increase the pathlength of the laser beam reflected from M1, interference fringes were generated at a spatial frequency directly related to the pathlength difference. By adjustment, the spatial frequency was set to 1.0 c deg^-1 ^which is readily detected for peripheral viewing at our eccentricities [26]. The contrast of the display was varied by rotation of the polarizer P4, since contrast is proportional to sin^2^θ where θ is the angle of rotation from the position of zero contrast. The intensities of the laser beam and background beam were equated using a UDT S370 Optometer at the position of the eye. Likewise, the intensities of the 4 quadrants were also equalized. The final viewing intensity was determined psychophysically to be 3.2 log units above foveal threshold.

### Contrast threshold determinations

Prior to the determinations, each subject or patient was given a standard explanation with the aid of diagrams as to the nature of the test, which was then carried out under standardized subdued illumination. The subject or patient viewed the display through the Maxwellian lens without external refraction. With the direction of gaze determined by the central fixation spot, the subject or patient increased the contrast of the grating display in the peripherally-located truncated quadrant until the pattern became just visible and no more. This gives results for contrast thresholds similar to those for the more time consuming 50% of seeing [27]. The angle of rotation was recorded and readings repeated. First, the subject or patient had practice runs with the truncated quadrant in each of its 4 positions. This was then followed by 6 contrast threshold determinations for each of the 4 truncated quadrant positions for each eye in turn. The tests including the preliminary Snellen test and explanation took 20–40 min to complete. Repeat determinations in 2 normal subjects and one glaucoma patients on different days confirmed the consistency of the contrast threshold values. The determinations for the glaucoma patients were undertaken prior to the release of the visual field charts for analysis and the time interval between the tests ranged from the same day to 36 weeks (mean 64 ± 55 S.D. days).

### Data analysis

Statistical analyses were undertaken with the Minitab statistical package [28]. The basic method of analysis involved the calculation of 95% prediction limits for the contrast thresholds and for the inter-ocular differences in contrast threshold between companion normal eyes, against which the results for the glaucoma patients were compared. After conversion of the data to cumulative probabilities, the sensitivity (the percentage of correctly classified glaucoma patients) was plotted against 1 – specificity (the percentage of incorrectly classified normal subjects) to obtain the Receiver Operating Characteristic (ROC) curve [29,30]. The area under the ROC curve is a measure of the sensitivity over a range of criterion levels and varies from 0.5 when there is an equal likelihood of glaucoma patients and normal subjects exceeding the specified criterion to 1.0 when there is perfect discrimination of glaucoma patients from normal subjects.

Comparisons between the mean contrast threshold for each truncated quadrant against visual field loss were undertaken by linear regression analysis after quantifying the visual field loss from the patient's Friedmann visual field chart [31]. A template containing an aperture corresponding to the dimensions of the truncated quadrant was laid over the Friedmann visual field chart and the following scores allocated to each point tested according to whether the point was visible through the applied neutral density filter: 3 – densest filter appropriate to age (usually 1.2 log units attenuation), 2 – next densest filter (usually 0.8 log units attenuation), 1 – no filter and 0 – not visible. The summed values were expressed as a percentage of the maximum possible score for that truncated quadrant and the difference from 100% was the visual field loss score. This was repeated for each of the 4 positions shown in Figure 2 and no allowance was made for the presence of the blind spot in the temporal quadrant. From the regression analysis, the value of *R*^2^, the coefficient of determination, was obtained. This gives as a percentage the amount of the variation in contrast threshold against visual field loss score which is accounted for by the line of best fit (regression line); so *R*^2 ^ranges from 0% when the data are randomly distributed to 100% when the data points fall exactly on the regression line. The value of *R*^2 ^is amenable to statistical testing and significance was taken as *p *< 0.05.

## Results

### Control group

The contrast thresholds for the 64 normal eyes were normally uniformly low for each of the superior, temporal, inferior and nasal truncated quadrants with no significant differences between the mean values of the 4 quadrants (*p *= 0.81). The mean contrast threshold and the 95% upper prediction limit were then calculated for the 4 truncated quadrants for the normal eyes of the group of 8 subjects together with one eye chosen at random from the remaining 28 subjects to give equal numbers of left and right eyes. The contrast threshold values for the group conformed closely to a normal distribution and had a mean value of 0.027 ± 0.011 S.D. contrast units, giving a 95% upper prediction limit of 0.045 contrast units. Inter-ocular comparisons were made between the mean values for left and right eyes in the group of 28 subjects with 2 normal eyes. The mean of the modulus of the difference was 0.0029 ± 0.0020 S.D. contrast units, giving a 95% upper prediction limit of 0.0060 contrast units. These upper prediction limits were then used to assess the contrast thresholds of the glaucoma patients and of the abnormal eyes of the non-glaucoma group.

The Humphrey Central 24-2 test for the 40 normal eyes in 23 subjects (the remaining 6 eyes had non-glaucomatous abnormalities), missed 5 blindspots, gave mean deviations outside normal limits in 8 eyes and gave abnormal or borderline results with the glaucoma hemifield test in 12 eyes. In all cases, the abnormal results were explained by drooping eyelids, obstruction by spectacles or trial frames and subject error, which are common hazards of automated perimetry [32].

### Glaucoma patients

**Figure 3:**
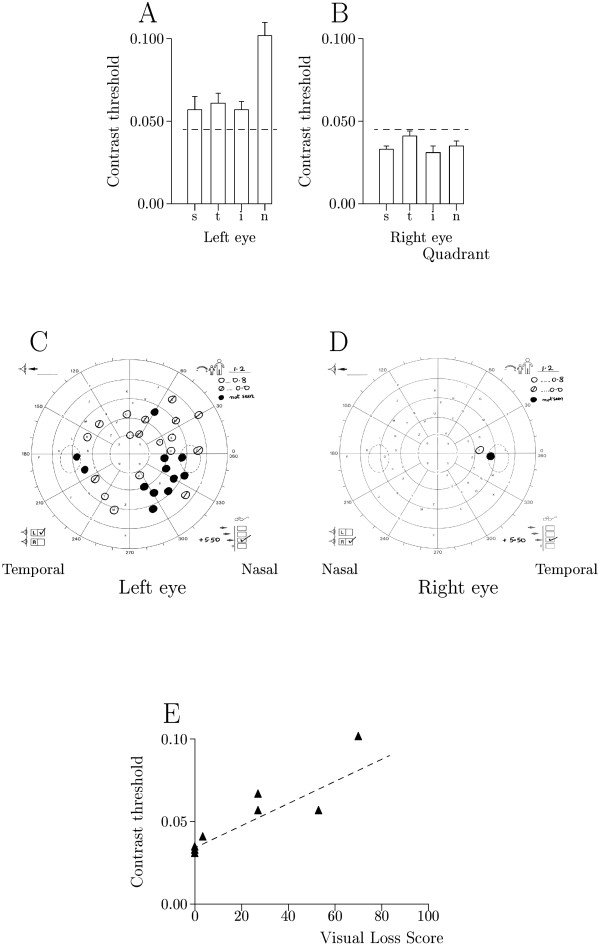
Patient 9 (Table 1) A & B Histograms of contrast threshold (mean S.E.M.) for superior (s), temporal (t), inferior (i) and nasal (n) truncated quadrants in the left and right eyes showing upper 95% prediction limit of 0.045 contrast units as broken horizontal line. Contrast thresholds were abnormal in the left eye. C & D Friedmann visual fields showing: unmarked letters-normal detection (1.2 logarithmic units attenuation), reduced sensitivity (○ 0.8 logarithmic units attenuation and ø zero attenuation) and ● zero detection. The main visual field loss was in the inferior hemifield of the left eye. E Contrast threshold against visual field loss score taken from Friedmann charts for each truncated quadrant, showing best fitting relationship as broken line. Relationship was significant: *R*^2 ^= 85%, *p *= 0.001.

**Figure 4:**
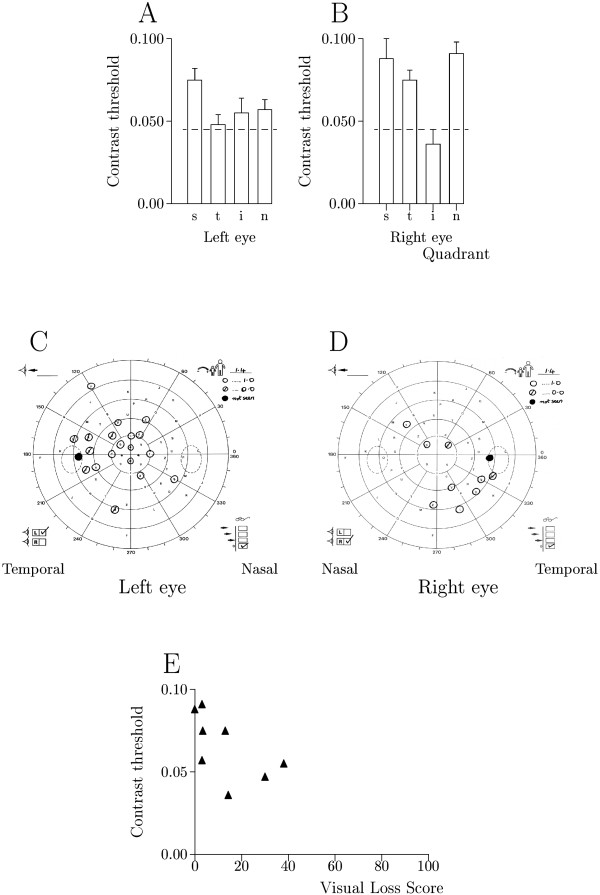
Patient 6. Same conventions as in Figure 3 but with ○ as 1.0 logarithmic units attenuation. A & B Contrast thresholds were abnormal in left and right eyes. C & D Visual field loss was diffuse in the left eye and occurred in inferior temporal quadrant of right eye. E Relationship between contrast threshold and visual field loss score was not significant: *R*^2 ^= 25%, *p *= 0.12.

The first comparison was to determine the extent to which the contrast threshold for a truncated quadrant exceeded the upper prediction limit of 0.045 contrast units, irrespective of the location of the quadrant. Abnormally high values occurred in 29 out of 33 glaucomatous eyes (87%) and, on a patient by patient basis, in 20 out of 21 patients (95%). There were no apparent differences among the different types of glaucoma (Table 1). Examples of patients with elevated contrast thresholds are shown in Figures 3 &4.

Second, a comparison was made of the modulus of the inter-ocular difference of the mean contrast threshold for left and right eyes against the upper prediction limit of 0.0060 contrast units. This was abnormally high in 18 out of 20 glaucoma patients (90%) for whom the comparison was possible, including the patient with bilateral normal contrast thresholds. However, when identification was made on the basis of the combination of either an abnormal contrast threshold or abnormal inter-ocular difference, 100% correct identification was achieved. The optimal sensitivity and specificity and the area under the ROC curve are given in Table 2 which also shows data for tests of contrast threshold restricted to the superior and inferior truncated quadrants and to the nasal and temporal truncated quadrants. Both these assessments show reduced sensitivity compared with testing of all 4 truncated quadrants. It is thus clear contrast threshold elevations, especially in combination with intra-ocular differences were effective in detecting glaucoma.

### OHT patients

In the 5 OHT patients, contrast thresholds were elevated in 4 out of 10 eyes, corresponding to 2 patients, with no differences between those with and without pathological cupping of the optic disc. The inter-ocular difference was elevated in 3 out of 5 OHT patients. Follow-up of the OHT patients over the next 12 months showed that those with normal contrast thresholds retained normal visual fields while the 2 patients with elevated thresholds developed marked visual field loss in both eyes (Table 1).

### Comparison of locations of contrast threshold elevation and visual field loss

Our main assessment was to determine whether those parts of the visual field which showed abnormally high contrast thresholds also corresponded to the location of the visual field loss. This was undertaken by regression analysis of contrast threshold against the corresponding visual field loss score for each of the truncated quadrants in both eyes. In order to achieve a reasonable distribution of data points, patients were required to have a range of visual field loss scores in excess of 20% together with an absence of visibility in no more than one of the truncated quadrants. This provided 18 glaucoma patients whose results are shown in Table 1. Contrast threshold and visual field loss were strongly related in 5 patients (*R*^2 ^= 55–85%, *p *< 0.05) as illustrated by Figure 3, showed a semblance of s direct relationship in 4 patients (*R*^2 ^= 46–49%, 0.05 <*p *< 0.10) and showed an absence of a relationship in 9 patients (*R*^2 ^= 0–26%, *p *> 0.1) as illustrated by Figure 4. There was no correlation between the form of the relationship and the type of glaucoma (Table 1). We also repeated the analysis in 8 patients with minimal visual field loss in one eye for which a single mean value was calculated (*i.e*. giving *n *= 5 for regression analysis). The *R*^2 ^values were unaffected in the new analysis (*p *= 0.84, paired *t*-test) and a significant or borderline relationship was still present in 5 patients.

### Control subjects with abnormal fellow eye

There were 8 subjects in the non-glaucoma control group with an abnormality of one eye. In the subject with the retinal scar, the contrast thresholds were elevated in the truncated quadrant containing the scar as confirmed by the Humphrey Visual Field. On the other hand, the contrast thresholds for the subjects with repaired retinal detachment, mild cataract and solar damage and for 2 amblyopes were normal. The third amblyope had abnormally high contrast thresholds while the subject with macular degeneration was unable to fixate with that eye.

## Discussion

Contrast thresholds were readily measured with our Maxwellian view interferometer in normal subjects, even in the presence of refractive errors as large as -7.00 DS and +5.00 DC, which is a positive advantage over externally viewed displays which depend on the accuracy of the refraction and factors like spectacle slippage or obstruction by the edges of frames. In a limited number of cases, contrast thresholds were readily obtained in the presence of minor lens opacities and in amblyopes, though the method is impracticable in cases where fixation cannot be achieved. Generally, the contrast thresholds of normal subjects were uniformly low. While evidence of the blind spot in the form of elevated contrast thresholds in the temporal truncated quadrant occurred infrequently, this was probably the result of stimulation of a large area of retina compared with the well circumscribed extent of the blind spot of which there is a perceptual lack of awareness [33]. The use of relatively large truncated quadrants may suggest intuitively that localisation of visual loss was being sacrificed though there is evidence to suggest that this is not the case. Threshold values between neighbouring test points tend to be correlated with the result that the central visual field is organized into discrete clusters or sectors which are related to the projection of retinal ganglion cell axons [34,35]. Another issue is the variation in the number of cycles between superior/inferior and nasal/temporal truncated quadrants. We chose the stimulus spatial frequency to be 1.0 c deg^-1 ^to ensure high sensitivity in the peripheral retina [26] and a number of cycles which did not limit sensitivity, at least for foveal viewing [36]. It is conceivable that, at a peripheral location, differences in the number of cycles may affect contrast sensitivity though such an effect is likely to be small [37]. Frequency Doubling Technology (FDT) perimetry employs stimulus fields not dissimilar to those employed in the present study though the grating pattern is phase modulated at a high temporal frequency to produce an illusory doubling of the spatial frequency [38]. Reduction of the dimension of the square stimulus field from 10° to 4° produced only a modest increase in contrast threshold [39]. These results are thus not inconsistent with the absence of a significant differences between the contrast thresholds for the different truncated quadrants.

In terms of correct identification of our glaucoma patients, contrast thresholds were elevated in one or more truncated quadrants in all but one of our 21 patients while inter-ocular differences were elevated in all but 2 out of 20 patients, which is consistent with previous studies [4,15-18,21]. These elevations were present irrespective of the type of glaucoma, which is consistent with a common mechanism for ganglion cell loss in chronic glaucoma [40]. In terms of detection of glaucoma, our method has performed soundly with an area under the ROC curve of 0.956 which compares well with other methodologies employed in the early detection of glaucoma *viz*. short wave length automated perimetry (SWAP), frequency doubling technology, resolution acuity perimetry, detection acuity perimetry and temporal modulation perimetry [41] and considerably better than for contrast sensitivities with central viewing [42]. Of especial interest was the performance of our test with the OHT patients. Initially, it seemed that two patients would be incorrectly diagnosed in both eyes which had markedly elevated contrast thresholds; however within the next 12 months they each developed appreciable visual field loss in their eyes (Table 1). While the sample size is small, it indicates a potential predictive function which has also been described for FDT, high-pass resolution perimetry and SWAP [43,44]. A further improvement in the area under the ROC curve to 0.993 occurred when the assessment of contrast thresholds was combined with that of the inter-ocular difference in contrast threshold. This is a simple extension of the analysis which uses pre-existing data and its value has been remarked upon previously [38].

The central aim of our study, however, was whether the contrast threshold results reflected the amount of visual field loss in the same region of the visual field. While there was a reasonable correspondence in 50% of our 18 glaucoma patients (Figure 3), in the other half of the patients, there was a marked mismatch so that some patients had normal contrast thresholds in quadrants showing high visual field loss while others had elevated contrast thresholds in seemingly normal parts of the visual field (Figure 4). We are confident that the results were not caused by abrupt shifts of visual field loss between quadrants since the visual fields were stable over the 2 years preceding our tests and indeed subsequent to our tests. While automated perimetry is widely used to ascertain visual field loss, our preference was firmly for the Friedmann test on the basis that it was less disconcerting to elderly patients and is consistent with the transferability of visual field data between different machines [45]. While our identification of the misalignment with visual field loss relates to the detection of a stationary grating pattern, allusions have also been made to FDT error scores which occurred in the hemifield opposite to that containing the visual field loss [46,47]. This lack of correspondence between contrast threshold elevation and visual field loss can reasonably be attributed to the different attrition rates for the different modalities of visual function in different individuals which is well known in glaucoma [12,38,48-53]. To this, we can further add that different modalities, at least in terms of light detection and contrast detection, are affected differently in different parts of the retina. The sentiment has been expressed that it does not matter whether two tests do not agree so long as the disease is detected during screening [47]. To an extent this is understandable though it would clearly be desirable to have a rational basis for whatever testing regime is adopted. One possible explanation is that conventional perimetry detects light detection defects [54] while tests like FDT, SWAP and contrast threshold measurements detect different manifestations of ganglion cell damage [40,41]. Certainly there are several tests, particularly FDT and SWAP, which could be used profitably alongside visual field testing [48] and, to these, contrast threshold determinations in the peripheral visual field may be added.

## Conclusion

Our results therefore do confirm that contrast thresholds in response to sinusoidal grating patterns presented to peripheral regions of the visual field are invariably abnormally elevated in cases of glaucomatous visual field loss. The regions of threshold elevations, however, did not map onto the regions of visual field loss, which indicates contrast threshold testing cannot be considered as a substitute for visual field tests. It may however provide valuable supplementary information which may be obtained simply and rapidly with the type of interferometric apparatus we have described.

## Competing interests

The author(s) declare that they have no competing interests.

## Authors' contributions

JLJ carried out the clinical examination of the patients. The scientific experiments were carried out and analyzed by CMT, JSM and JDM who also undertook the construction of the apparatus and drafting of the manuscript which all authors approved.

## Pre-publication history

The pre-publication history for this paper can be accessed here:



## Figures and Tables

**Table 1:** Clinical data for glaucoma and OHT patients

Pat no.	Age (yr)	LE Condition	LE VA	LE VF loss	LE CT_*max*_	RE Condition	RE VA	RE VF loss	RE CT_*max*_	I-O diff	R^2^
1	72	POAG	6/5	74%	0.051	POAG	6/4	7%	0.023	0.025	79%*
2	73	POAG	6/6	60%	0.098	POAG	6/7.5	51%	0.168	0.045	0%°
3	67	POAG	6/6	4%	0.099	NPG	6/9	53%	0.107	0.004	48%^+^
4	76	POAG	6/6	7%	0.075	POAG	6/7.5	44%	0.078	0.001	1%°
5	71	POAG	6/6	36%	0.142	POAG	6/6	30%	0.067	0.045	0%°
6	66	POAG	6/6	17%	0.075	POAG	6/12	7%	0.091	0.014	-25%°
7	77	POAG	6/24	55%	0.055	POAG	6/6	67%	0.044	0.012	14%°
8	68	POAG	6/6	23%	0.069	normal	6/5	4%	0.049	0.019	55%*
9	75	POAG	6/9	44%	0.102	normal	6/5	1%	0.041	0.034	85%*
10	72	normal	6/6	2%	0.023	POAG	6/9	42%	0.031	0.006	47%^+^
11	37	POAG	6/18	14%	0.089	blind	nil	100%	n/s	na	na
12	83	POAG pxf	6/9	1%	0.066	POAG pxf	6/9	51%	n/s	na	na
13	83	POAG pxf	6/18	69%	0.058	POAG pxf	6/12	46%	0.074	0.025	-24%°
14	74	POAG pxf	6/5	26%	0.067	OHT	6/6	3%	0.056	0.008	9%°

15	73	NPG	6/5	17%	0.055	NPG	6/5	47%	0.033	0.014	0%°
16	76	NPG	6/9	18%	0.150	NPG	6/12	47%	0.060	0.021	46%^+^
17	73	NPG	6/9	53%	0.048	normal	6/9	4%	0.027	0.010	49%^+^
18	54	RD	6/12	2%	0.046	NPG	6/5	41%	0.083	0.023	85%*

19	61	PCACG	6/9	11%	0.078	ACG	6/9	3%	0.072	0.012	na
20	79	PTG	6/9	55%	0.147	normal	6/9	6%	0.077	0.055	26%°
21	55	normal	6/6	7%	0.044	SIG	6/6	52%	0.058	0.017	73%*

22	70	OHT	6/5	0%(2%)	0.031	OHT	6/5	0% (6%)	0.018	0.002	na
23	78	OHT	6/5	0% (7%)	0.022	OHT	6/9	3% (8%)	0.033	0.0064	na
24	57	OHT	6/5	6% (13%)	0.054	OHT	6/5	4%(13%)	0.051	0.0006	na
25	62	OHT	6/5	2% (2%)	0.034	OHT pdc	6/12	4% (6%)	0.042	0.010	na
26	72	A OHT pdc	6/60	8% (29%)	0.155	OHT pdc	6/12	8% (35%)	0.059	0.068	na

Pat no. Patient number, LE left eye, RE right eye, A amblyopic, CT_*max *_highest contrast threshold value (to be compared against the upper prediction limit of 0.045), I-O diff inter-ocular difference (to be compared against the upper prediction limit of 0.006), NPG normal pressure glaucoma, OHT ocular hypertensive, na not applicable, n/s not seen, PCACG primary chronic angle closure glaucoma, pdc pathological cupping of optic disc, POAG primary open angle glaucoma, PTG post-traumatic glaucoma, pxf pseudoexfoliation, RD retinal detachment, SIG sarcoid-induced glaucoma, VA visual acuity, VF visual field. Negative *R*^2 ^values denote inverse relationship. *p < 0.05, ^+^0.05 <*p* < 0.10, °*p* > 0.10. Some eyes denoted normal show visual field loss due to the blind spot while some glaucomatous eyes showing minimal visual loss had additional loss either more peripheral or more central to the truncated quadrant. OHT patients also show visual field loss score in parenthesis at 12 months follow-up examinations.

**Table 2:** Signal detection data

Criterion	*n*	Sensitivity	Specificity	Area under ROC
CT_*max *_by eye	33	93.9%	91.0%	0.956
CT_*max *_S/I	33	87.8%	91.0%	0.953
CT_*max *_N/T	33	81.8%	91.0%	0.923
CT_*max *_by patient	21	90.4%	95.0%	0.954
I-O diff	20	89.5%	85.7%	0.897
CT_*max *_or I-O diff	20	95.0%	100%	0.993

CT_*max *_highest contrast threshold value, S/I superior and inferior truncated quadrants, N/T nasal and temporal truncated quadrants, I-O diff inter-ocular difference.
